# Predictive Capabilities of Human Leukocyte Antigen-G and Galectin-13 Levels in the Amniotic Fluid and Maternal Blood for the Pregnancy Outcome

**DOI:** 10.3390/medicina60010085

**Published:** 2024-01-01

**Authors:** Márió Vincze, János Sikovanyecz, András Molnár, Imre Földesi, Andrea Surányi, Szabolcs Várbíró, Gábor Németh, János Sikovanyecz, Zoltan Kozinszky

**Affiliations:** 1 Department of Obstetrics and Gynecology, University of Szeged, H-6725 Szeged, Hungary; vincze.mario92@gmail.com (M.V.); janossikovanyecz@gmail.com (J.S.J.); molnar.andras@med.u-szeged.hu (A.M.); gaspar-suranyi.andrea@med.u-szeged.hu (A.S.); varbiroszabolcs@gmail.com (S.V.); nemeth.gabor@med.u-szeged.hu (G.N.); dr.sikovanyecz@gmail.com (J.S.); 2 Department of Laboratory Medicine, University of Szeged, H-6720 Szeged, Hungary; foldesi.imre@med.u-szeged.hu; 3 Department of Obstetrics and Gynecology, Danderyd Hospital, 182 88 Stockholm, Sweden

**Keywords:** soluble human leukocyte antigen-G, placental protein-13, serum, amniotic fluid, sonography

## Abstract

*Background and Objectives*: Increasing evidence points to the significant role of the angiogenic factor levels in screening for pregnancy outcome. To examine the potential relationship between concentrations of placental protein 13 (PP13) and soluble human leukocyte antigen-G (sHLA-G) in maternal serum and amniotic fluid at 16–23 weeks of gestation and the sonographic features of pregnancy as well as pregnancy outcome. *Materials and Methods*: PP13 and sHLA-G in serum and amniotic fluid, fetal biometrical data, and placental volume and perfusion indices were determined in 71 euploid, singleton pregnancies. *Results*: The serum sHLA-G level exhibits a negative correlation with the serum PP13 level (r = −0.186, *p* < 0.001) and a positive correlation with the sHLA-G level in amniotic fluid (r = 0.662, *p* < 0.001). A significant correlation was found between serum sHLA-G level and placental volume (r = 0.142, *p* < 0.05) and between amniotic sHLA-G level and placental perfusion (r = −0.450, *p* < 0.001). A low amniotic PP13 level significantly predicted the birth weight (r = −0.102, *p* < 0.05), the duration of pregnancy (r = −0.155, *p* < 0.05), and the fetal abdominal circumference (r = −0.098, *p* < 0.05). *Conclusions*: PP13 assayed in amniotic fluid might be a potential marker of fetal growth, and sHLA-G can be an adjunct modality reflecting placental sonographic parameters.

## 1. Introduction

Placental growth includes extensive vasculogenesis and angiogenesis controlled by multifunctional angiogenic factors, which are expressed by trophoblasts, immune cells, and endothelial cells and also secreted into the maternal circulation [1,2]. Human leukocyte antigen-G (HLA-G) is a member of the class Ib histocompatibility family, which is expressed in the placenta [3]. Soluble (s)HLA-G isoforms include HLA-G1 and HLA-G5-7. The sHLA-G isoforms are detectable in both extravillous and villous cytotrophoblasts, as well as in the chorion membrane and syncytiotrophoblasts [3,4]. The sHLA-G isoforms play critical roles in trophoblast invasion, remodelling of spiral arteries, fetal development, and materno-fetal immune tolerance [3,5]. Increasingly, findings support the role of HLA-G in complications associated with pregnancy. The level of total soluble and membrane-bound HLA-G decreased significantly in cases of preeclamptic placenta and maternal serum compared with placenta and maternal serum of pregnant women with no relevant complications in [6,7]. An increased level of HLA-G5 in the placenta [8] and in maternal serum [9] was observed in preeclampsia (PE), whereas a diminished sHLA-G1 level was described in PE [9] in the third trimester. Decreased sHLA-G level and reduced membrane-bound HLA-G protein expression were linked to recurrent miscarriage [10].

Placental protein-13 (PP13) is a member of the galectin family and is synthesized in syncytiotrophoblasts [11]. PP13 promotes the migration and differentiation of trophoblasts and is implicated in the vascular remodelling of spiral arteries in early pregnancy. Moreover, it contributes to immunoregulation by inducing apoptosis of maternal T cells [12,13]. PP13 can predict preeclampsia in the first trimester [14,15]. Maternal serum PP13 levels are lower in pregnancies complicated by gestational diabetes mellitus (GDM) [16]; however, diminished levels of PP13 were found in non-complicated pregestational diabetes mellitus [17].

Both sHLA-G and PP13 can be detected in maternal blood [14,15,16,18,19,20,21,22] and amniotic fluid [19,23,24,25]. The interrelation of the fetoplacental growth during gestation and the levels of these angiogenic factors in maternal serum and amniotic fluid have not been investigated yet. Therefore, this study aims to determine sHLA-G and PP13 protein levels related to sonographic parameters of the fetus and placenta during mid-trimester as well as to perinatal outcome.

## 2. Materials and Methods

### 2.1. Study Design

A prospective, cross-sectional cohort study was conducted in pregnant women undergoing amniocentesis at the Department of Obstetrics and Gynecology, University of Szeged, Hungary between January 2019 and December 2020. During the study period, all singleton pregnancies with increased risk of chromosomal abnormality, where amniocentesis (AC) was performed between 16 + 0 and 22 + 6 weeks of gestation, were recruited into our study. The indications for AC were increased nuchal translucency (NT) at first trimester scan (≥2 MoM for gestational age) (n = 20), chromosome aberration or gene disorder concerning the previous pregnancy (n = 11), and advanced maternal age (n = 40).

Exclusion criteria of the study were identified as follows: multiple pregnancies; fetal or neonatal structural or genetic anomaly; improper localization of the placenta for sonography (placenta praevia, posterior placenta); pathological placentation (placenta accreta spectrum); self-reported drug, alcohol, caffeine, or nicotine abuse or exposure to circulatory medication (oxerutins, calcium dobesilate); and systemic disease (e.g., any type of pregestational diabetes mellitus, autoimmune disease, vasculitis, hemophylia, thrombophylia, HIV infection).

Women with complications during late pregnancy (GDM treated with diet (n = 12), hypertension-related diseases (n = 11), small for gestational age (SGA) at delivery (n = 4), large for gestational age at delivery (n = 10)) were not excluded from the study.

The study protocol was approved by the Clinical Research Ethics Committee of the University of Szeged (the reference number: 09/2017 and the date of approval: 10 February 2017). The study was carried out according to the Principles of the Declaration of Helsinki. We obtained written informed consent from all participants.

### 2.2. Conventional 2-Dimensional (2-D) Sonographic Examinations

All pregnancies were dated by using the measurement of crown–rump length (CRL) at nuchal screening. NT and anatomic assessment between 11 + 0 and 13 + 6 weeks were performed by utilizing conventional methods. Ultrasound examination took place before measuring AC to determine the number of fetuses, fetal biometry, fetal anomalies, placental location, and the amount of amniotic fluid. Fetal weight was estimated according to the method of Hadlock et al. [26] after measuring the necessary sonographic parameters (biparietal diameter, head circumference, abdominal circumference, and femur length). Estimated fetal weight percentile was calculated according to the local standards [27]. The ultrasound investigations were conducted by J. S., A. S. and A. M.

### 2.3. Volume Acquisition

The images used for the determination of placental volume and 3-dimensional power Doppler (3-DPD) indices were acquired at the time of the visit. All 3D scans were performed by A. S. Voluson 730 Expert ultrasound machine (GE Medical Systems, Kretztechnik GmbH & Co OHG, Tiefenbach, Austria) equipped with a multifrequency probe (2–5 MHz), which was used to acquire all images. Each sample was examined using 3D rendering mode, in which the color and gray value information was processed and combined to give a 3D image (mode cent; smooth: 4/5; FRQ: low; quality: 16; density: 6; enhance: 16; balance: 150; filter: 2; actual power: 2 dB; pulse repetition frequency: 0.9). We used fast, low-resolution acquisition to avoid any kinds of artifacts. The 3D static volume box was placed over the highest villous vascular density zone at the umbilical cord insertion [28]. Each image was recovered from the disc in succession for processing. We recorded one sample from each patient during gestation.

### 2.4. Determination of Power Doppler Indices

The stored volumes were further analyzed using the virtual organ computer-aided analysis (VOCAL) program pertaining to computer software 4DView (GE Medical Systems, Zipf, Austria, version 10.4) by the same expert in 3D analysis (A.S.). The image used for recovering from the hard disc was captured and processed using the multiplanar system. The spherical sample volume was consistently 28 mL. The VOCAL program automatically calculated the gray- and color-scale values from the acquired spherical sample volume in a histogram in all cases. The combined use of power Doppler with three-dimensional ultrasound provides the possibility of quantifying blood in motion within a volume of interest. Three indices were calculated, namely the vascularization index (VI), flow index (FI), and vascularization flow index (VFI), as estimates of the percentage of the volume filled with detectably moving blood. VI (expressed as a percentage) is the proportion of color voxels in the studied volume, representing the proportion of blood vessels within the tissue. FI (expressed as a scale of 0–100) is the average value of all color voxels, representing the average power Doppler amplitude within blood vessels. VFI (expressed as a scale of 0–100) is the average color value of all gray and color voxels, a product of the number of color voxels as a percentage and the relative amplitude of these voxels [29,30].

The intra-observer errors were evaluated by repeated measurements of 3-DPD indices at initiation of the study. The intra-class correlation coefficients for all Doppler indices were excellent (0.99) in case of all indices.

### 2.5. Amniocentesis Procedure

The patients were informed about the procedure and possible complications before a consent form was signed prior to the procedure. All procedures were performed by the same operator expert (J.S.) at the outpatient unit, who followed the standard protocol. A local antiseptic was applied to the skin. A 22-gauge spinal needle was inserted under continuous ultrasound guidance, and needle insertion through the placenta was avoided. Amniotic fluid (8–10 mL) was aspirated, and the first 2 mL of each sample were discarded to prevent contamination with maternal cells. Blood-contaminated amniotic fluid was not utilized. Fetal heart rate was evaluated after the procedure, and no stillbirth or premature rupture was observed. Following amniocentesis, anti-D immunoglobulin was administered when it was necessary.

### 2.6. Samples

Amniotic fluid and maternal venous blood were collected from each patient at the time of amniocentesis. Blood samples were centrifuged at 3400 rpm for 15 min. Serum and amniotic fluid samples were stored at −80 °C until assay.

### 2.7. Enzyme-Linked Immunosorbent Assay (ELISA)

Human PP13 and human sHLA-G in maternal serum and amniotic fluid were determined by ELISA. The laboratory staff members who performed the assays were blinded to the pregnancy outcomes, and the clinician recruiting women did not participate in analyzing the samples.

The concentration of sHLA-G was measured using the kits from Elabscience Biotechnology Corporation (Houston, TX, USA). The sensitivity of the assay was 0.38 ng/mL. The intra- and inter-assay coefficients of variation were <10%, according to the manufacturer.

Human PP13 levels were determined by Cusabio kits (Wuhan Huamei Biotec Co., Ltd. Wuhan, China). The sensitivity of the assay was <3.9 pg/mL. The intra- (<8%) and inter- (<10%) assay coefficients of variation were according to the manufacturer.

### 2.8. Data and Statistical Analysis

Statistical analyses were performed using SPSS version 23 (IBM) (SPSS Inc, Chicago, IL, USA). Continuous variables were expressed as mean ± standard deviation (SD), and categorical variables were expressed as numbers and percentages. The relationship between the level of angiogenic factors (sHLA-G and PP13) and other continuous variables was assessed using Pearson’s correlation coefficients and regression analyses. Multiple linear regression was adjusted for maternal age, BMI at the time of genetic consultation, number of previous pregnancies, and gestational age at the time of amniocentesis, as these factors determine the actual placental volume and fetal weight. Correlation coefficients (B) were calculated for both univariate and multiple linear regression, whereas standardized coefficients (ß) were given for univariate analyses and semi-partial correlations (r) for multivariable regression. Independent sample *t*-tests were used to determine whether the angiogenic factor levels in body fluid were different in complicated pregnancies vs. healthy subjects. The paired samples *t*-test was applied to analyze the differences between serum and amniotic levels of the analytes. The two-tailed statistical significance level was set at 5%, and *p*-values were adjusted using the Holm–Bonferroni correction for multiple comparisons.

## 3. Results

Demographic characteristics of the group are shown in Table 1.

As expected, amniocentesis has been proposed mostly for pregnant women of advanced age (mean 34.52 years). Women who participated in this study had a mean body mass index (BMI) of 26.98 kg/m^2^, and approximately one-third (32.39%) of the participants were primiparous. The mean gestational age during amniocentesis was 18 + 2 weeks. Sonographic parameters are presented in Table 2.

The mean percentiles of the anthropometric measurements were near the median. We found a relatively large variation of intrauterine placental volume and perfusion indices.

Table 3 provides an overview of the levels of angiogenic factors in amniotic fluid samples. The mean concentrations of angiogenic factors were as follows: 8.68 pg/mL for PP-13 in amniotic fluid and 204.23 pg/mL for PP-13 in serum, 55.89 ng/mL for sHLA-G in amniotic fluid and 55.84 ng/mL for sHLA-G in serum.

**Figure 1:**
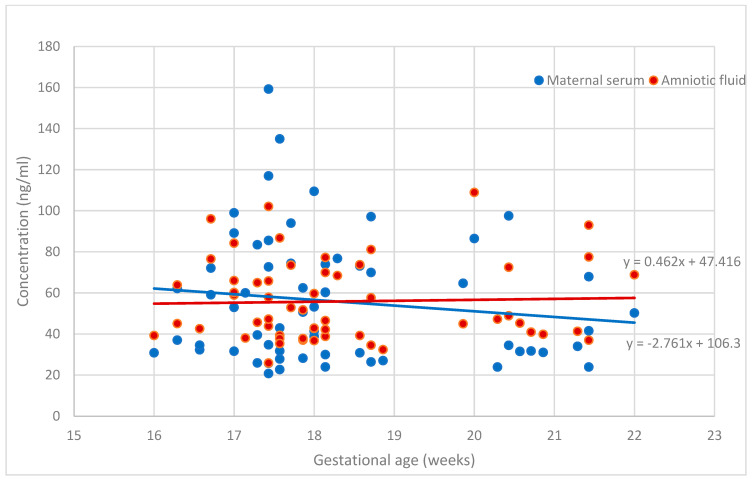
The secretion of sHLA-G in maternal serum and amniotic fluid.

**Figure 2:**
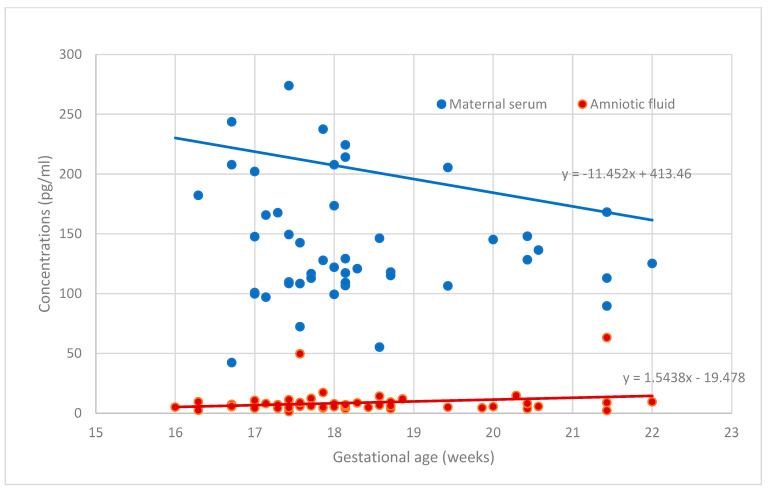
The secretion of PP13 in maternal serum and amniotic fluid.

Figure 1 and Figure 2 demonstrate the placental angiogenic factor levels in the body fluids according to gestational age. The sHLA-G levels were steady during this gestational period. The PP13 concentration in the amnion was unchanged between 16 + 0 and 22 + 6 weeks of gestation, while the concentrations of PP13 in the serum slightly decreased, but the reduction was below statistical significance. The concentrations in serum and amniotic fluid were significantly different for PP13 (*p* < 0.001) but not for sHLA-G (*p* > 0.05).

When regrouping was performed and uncomplicated pregnancies were compared to those complicated by hypertensive disorders during pregnancy, diabetes in pregnancy, and small or large for gestational age at pregnancy, no placental biomarkers exhibited a significant difference with regard to any of the measured factors in serum and amniotic fluid (data will be published later).

**Figure 3:**
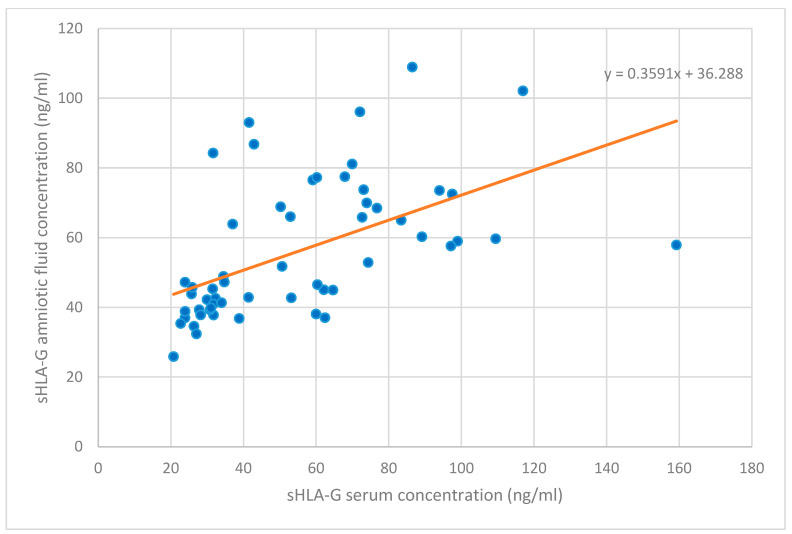
The correlation between sHLA-G concentrations in serum and amniotic fluid (*p* < 0.001).

Table 4 displays the correlation to levels of angiogenic factors. An inverse correlation emerged between sHLA-G levels and PP13 levels in serum in univariate and multivariate analyses (r = −0.186, B = −0.07, ß^2^ = 0.14, and B = −0.07, r^2^ = 0.16, respectively), whereas sHLA-G levels in serum and amniotic fluid appeared to have a strong positive correlation with each other (univariate analysis: r = 0.662, B = 0.36, ß^2^ = 0.29, and multivariate analysis: B = 0.37, r^2^ = 0.30, respectively, Figure 3). No other correlations were found between angiogenic factor levels in serum and amniotic fluid.

Table 5 demonstrates the sonographic correlation to sHLA-G levels. The sHLA-G serum concentrations tended to be higher with larger placental volumes (univariate analysis: r = 0.142, B = 0.09, ß^2^ = 0.09, and multivariate analysis: B = 0.09, r^2^ = 0.06, respectively). There was a strong indirect correlation between VFI and sHLA-G levels in amniotic fluid (univariate analysis: r = −0.450, B = −1.90, ß^2^ = 0.14, and multivariate analysis: B = −2.00, r^2^ = 0.13, respectively).

The correlations between PP13 levels and sonographic features are displayed in Table 6. The PP13 concentration in amniotic fluid displayed a negative correlation to fetal weight (univariate analysis: r = −0.102, B = −0.01, ß^2^ = 0.07, and multivariate analysis: B = −0.01, r^2^ = 0.06, respectively) and gestational age at delivery (univariate analysis: r = −0.155, B = −2.64, ß^2^ = 0.15, and multivariate analysis: B = −3.02, r^2^ = 0.18, respectively). An inverse correlation was observed between the level of PP13 in amniotic fluid and fetal abdominal circumference at amniocentesis (univariate analysis: r = −0.098, B = 0.31, ß^2^ = 0.15, and multivariate analysis: B = 0.49, r^2^ = 0.09, respectively).

## 4. Discussion

Placental growth encompasses extensive vascularization in the chorionic villi, which is regulated by angiogenic factors [5,10,25,31]. Circulating angiogenic factors have been shown to be associated with fetoplacental growth [9,10,14,25,32] and pregnancy disorders [14,32]. Placenta-related disorders, especially fetal growth restriction, can lead to stillbirth, and some cases can be prevented by adequate antenatal care [33]. In this study, we observed that sHLA-G concentration in maternal serum and amniotic fluid is stable in the mid-trimester, despite the expansive increase in trophoblasts, fetal membrane epithelial and immune cells secreting sHLA-G proteins. The unchanged longitudinal concentration of sHLA-G in body fluids is in correlation with the results reported by others [9,23,32,34,35].

Moreover, there is a paucity in the literature concerning soluble HLA-G levels in amniotic fluid [7,24,36]. A new and interesting finding is that amniotic sHLA-G levels correlates highly to sHLA-G levels in maternal peripheral blood at the time of amniocentesis. The amniotic sHLA-G is mainly synthesized by villous trophoblasts and, therefore, is of fetal origin. The strong positive correlation of amniotic sHLA-G level to maternal serum level remains elusive [19]. One of the possible explanations may be that extravillous trophoblasts (EVT) produce sHLA-G both for maternal serum and amniotic fluid.

Furthermore, one of the principal results of the present study is that the sHLA-G level in maternal serum is a valuable marker of placental growth but not of fetal growth. This can be explained by the fact that an increased amount of EVT cells in a larger placenta can promote an elevated serum sHLA-G level in pregnant women. On the other hand, the elevated secretion of sHLA-G by maternal monocytes can be stimulated by the increased expression of antigens presented by a larger EVT volume [5,36].

Conversely, the sHLA-G level in the amnion had a negative correlation with the vascular perfusion of the placenta. We assume that sHLA-G secretion may be reactive to reduced blood flow and the vascular network in the expanding placenta as gestation advances, since sHLA-G facilitates the process of vasculogenesis [9]. The expansion of placental volume may outweigh the increase in capillary branching. In order to evaluate the placental perfusion, we used “Mercé-type sonobiopsy”, which is a reproducible and validated method [37,38,39], and by obtaining a representative sample of the placental tree, it is applicable throughout the entire pregnancy, unlike other methods [29], when the entire placenta needs to be visualized [40,41]. No significant correlation was found between fetal growth parameters measured by ultrasound examination and the corresponding level of sHLA-G in different body fluids. One study [9] found a statistically significant correlation between sHLA-G1 levels in maternal serum and neonatal birth weight and placental weight at delivery.

The importance of sHLA-G is supported by its lower serum level in PE, according to several reports [6,24,35], whereas a higher sHLA-G level in amniotic fluid as well as in maternal blood indicates the immune response to maternal infection, inducing preterm birth [7,32,36].

We found a small, non-significant decrease in PP13 concentration in maternal serum and a constant PP13 level in amniotic fluid between the 16th and 23rd weeks of gestation. In other studies, minimal elevation of serum PP13 concentration was reported during all trimesters [42,43,44,45]. Moreover, most of the studies on PP13 serum concentrations have focused on the prognostic performance in the field of pregnancy outcomes in the first trimester and at term [43]. Additionally, our interesting finding is that there is a decimal range difference between the levels in serum and in amniotic fluid, which was also reported by Sammar et al. [44] in both complicated (PE) and uncomplicated pregnancies during the third trimester. Like others [42,43,44,45], we have noticed a wide range of distribution of PP13 levels both in serum and amniotic fluid. Only two earlier studies have evaluated the secretion of PP13 into amniotic fluid [44,46]. Sammar et al. [44] concluded that the PP13 level measured in amniotic fluid that was derived from cesarean section is steady between 30 and 42 weeks of gestation, while a declining trend in the PP13 level can be described in PE between 26 and 36 weeks of gestation. Inconsistently, the amount of PP13 detected in amniotic fluid was twice as much as in serum samples in a small-scale study [46].

In our dataset, there was no difference in serum PP13 levels between women with pregnancy-related hypertensive disorders or SGA and controls. This was supported by Burger et al. [46], indicating that serum PP13 levels are lower in the first trimester in these types of complications, whereas the levels are higher in the third trimester, and the transition from the low to the high level occurs between the 16th and 20th gestational weeks [46], which includes the gestational range of our study. Several studies highlight the predictive value of serum PP13 concentration during the first trimester in developing preeclampsia [20,43,47], whereas no evidence has been raised for the corresponding tendency in the second trimester [48].

Our study revealed that PP13 levels in amniotic fluid in the second trimester can predict birth weight, which has not been previously proven. There is a reverse correlation between PP13 and birth weight. An additional finding is that PP13 levels in body fluids are not related to the size of the placenta. However, the opposite was confirmed by Sharawand et al. [49], who described that maternal serum PP13 level is associated with placental volume in the first trimester. On the other hand, it is unclear why amniotic PP13 levels prognosticate lower birth weight at delivery since it is secreted from placental trophoblasts and is involved in normal implantation and placentation [43].

The strength of our study is that a robust, large-scale amniocentesis study was performed in one center with respect to the prognostic efficacy of angiogenic biomarkers with reference to pregnancy outcome. The limitation of the study is that the samples were obtained from a high-risk population, and, hence, the generalizability of the study results is not possible for the whole population.

Our conclusion of particular value is that we can prove that maternal sHLA-G and PP13 concentrations in serum have a negative correlation to each other and sHLA-G levels in different body fluids have a strong positive correlation to each other. PP13 decreases slightly in maternal serum but not in amniotic fluid, whereas HLA-G is unchanged in both compartments in mid-pregnancy, as published by others. Amniotic PP13 might be used as a predictor of pregnancy outcome, while sHLA-G is a potent biomarker in placental development. This study also indicates that late complications during pregnancy are not associated with altered levels of sHLA-G and PP13 at amniocentesis.

## Figures and Tables

**Table 1:** Clinical and obstetrical data of women with amniocentesis (*N* = 71).

Maternal age (years) *	34.52 ± 5.78
Number of nulliparous women in the study **	23 (32.39)
BMI at the time of genetic consultation (kg/m^2^) *	26.98 ± 5.90
Gestational age at the time of amniocentesis (weeks) *	18.25 ± 1.42
Fetal weight at delivery (grams) *	3424.08 ± 538.63
Gestational age at delivery (weeks) *	39.09 ± 1.38

* Continuous variables displayed as mean ± standard deviation (SD). ** Categorical variables are presented in number and %.

**Table 2:** Ultrasound data at amniocentesis (*N* = 71) *.

Fetal biometry	
Head circumference (mm)	151.54 ± 16.18
Head circumference (percentile)	53.50 ± 30.79
Abdominal circumference (mm)	129.18 ± 17.07
Abdominal circumference (percentile)	51.63 ± 27.88
Femur length (mm)	27.04 ± 4.73
Femur length (percentile)	55.91 ± 30.52
Estimated fetal weight (grams)	243.20 ± 74.12
Estimated fetal weight (percentile)	52.92 ± 26.81
Placental sonography	
Placental volume (mm^3^)	227.36 ± 93.21
VI	14.11 ± 5.14
FI	44.97 ± 22.64
VFI	8.21 ± 3.63

VI: vascularization index, FI: flow index, VFI: vascularization flow index. * Continuous variables displayed as mean ± standard deviation (SD).

**Table 3:** Levels of angiogenic factors in samples of amniotic fluid and serum (*N* = 71) *.

PP-13 concentration in amniotic fluid (pg/mL)	8.68 ± 9.85
PP-13 concentration in serum (pg/mL)	204.23 ± 171.34
sHLA-G concentration in amniotic fluid (ng/mL)	55.89 ± 19.51
sHLA-G concentration in serum (ng/mL)	55.84 ± 30.50

PP-13: placental protein-13, sHLA-G: soluble human leukocyte antigen-G. * Continuous variables displayed as mean ± standard deviation (SD).

**Table 4:** Correlation between maternal and sonographic data and the levels of angiogenic factors in maternal serum and amniotic fluid (*N* = 71).

	sHLA-G Serum Level					sHLA-G Amniotic Fluid				
	Pearson r	Univariate Linear Regression		Multivariate Linear Regression		Pearson r	Univariate Linear Regression		Multivariate Linear Regression	
		B	β^2^	B	r^2^		B	β^2^	B	r^2^
PP13 in serum	−0.186 **	−0.07 **	0.14 **	−0.07 **	0.16 **	0.052	−0.03	0.08	−0.30	0.07
PP13 in amniotic fluid	−0.095	−0.55	0.03	−0.55	0.03	0.084	0.16	0.01	0.12	0.00
sHLA-G in amniotic fluid	0.662 **	0.36 **	0.29 **	0.37 **	0.30 **	-	-	-	-	-
	PP13 serum level									
	Pearson r	Univariate linear regression		Multivariate linear regression						
		B	β^2^	B	r^2^					
PP13 in amniotic fluid	0.111	0.84	0.00	2.50	0.01					

PP-13: placental protein-13, sHLA-G: soluble human leukocyte antigen-G. ** *p* < 0.001.

**Table 5:** Correlation between maternal and sonographic data and the levels of sHLA-G in maternal serum and amniotic fluid (*N* = 71).

	sHLA-G Serum Level					sHLA-G Amniotic Fluid				
	Pearson r	Univariate Linear Regression		Multivariate Linear Regression		Pearson r	Univariate Linear Regression		Multivariate Linear Regression	
		B	β^2^	B	r^2^		B	β^2^	B	r^2^
Clinical and obstetrical characteristics										
Maternal age	0.090	0.49	0.01	0.44	0.00	−0.106	−0.43	0.02	−0.48	0.01
BMI at the time of genetica consultation (kg/m^2^)	−0.012	0.01	0.00	−0.11	0.00	0.010	−0.17	0.00	−0.16	0.00
Fetal weight at delivery	0.094	0.01	0.03	0.01	0.03	0.083	0.01	0.01	0.01	0.02
GA at delivery	−0.163	−2.41	0.01	−2.15	0.01	0.056	0.90	0.00	0.73	0.00
GA at the time of amniocentesis (weeks)	−0.102	−2.76	0.02	−2.32	0.01	−0.041	0.46	0.00	0.26	0.00
Fetal sonography at the time of amniocentesis										
Head circumference (mm)	−0.047	−0.17	0.01	0.16	0.00	0.045	0.08	0.00	0.20	0.01
Abdominal circumference (mm)	0.112	0.11	0.00	1.20	0.08	0.204	0.36	0.10	0.99	0.11
Femur length (mm)	−0.120	−0.69	0.01	−0.30	0.00	0.119	0.85	0.05	−0.31	0.00
Estimated fetal weight (grams)	0.042	−0.00	0.00	0.17	0.02	0.175	0.06	0.06	0.18	0.05
Placental sonography at the time of amniocentesis										
Placental volume (mm^3^)	0.142 *	0.09 *	0.08 *	0.09	0.06	−0.043	0.00	0.00	0.01	0.00
VI	−0.114	0.12	0.00	−0.02	0.00	−0.256	−0.65	0.03	−0.62	0.02
FI	0.042	0.10	0.01	0.12	0.01	−0.090	0.11	0.02	0.15	0.03
VFI	−0.234	−1.00	0.02	−1.33	0.02	−0.450 **	−1.90 **	0.14 **	−2.00 **	0.13 **

sHLA-G: soluble human leukocyte antigen-G, BMI: body mass index, GA: gestational age, VI: vascularization index, FI: flow index, VFI: vascularization flow index. * *p* < 0.05; ** *p* < 0.001.

**Table 6:** Correlation between maternal and sonographic data and the levels of PP13 in maternal serum and amniotic fluid (*N* = 71).

	PP13 Serum Level					PP13 Amniotic Fluid				
	Pearson r	Univariate Linear Regression		Multivariate Linear Regression		Pearson r	Univariate Linear Regression		Multivariate Linear Regression	
		B	β^2^	B	r^2^		B	β^2^	B	r^2^
Clinical and obstetrical characteristics										
Maternal age (years)	0.057	3.30	0.01	2.44	0.00	0.076	−0.11	0.00	0.06	0.00
BMI at the time of genetic consultation (kg/m^2^)	0.114	2.87	0.01	2.79	0.01	0.084	−0.18	0.01	−0.17	0.01
Fetal weight at delivery (kg)	−0.174	−0.03	0.01	−0.04	0.01	−0.102 *	−0.01 *	0.07 *	−0.01	0.06
GA at delivery (weeks)	0.045	13.2	0.01	13.07	0.01	−0.155 **	−2.64 **	0.15 **	−3.02 **	0.18 **
GA at the time of amniocentesis (weeks)	−0.078	−11.45	0.01	−5.45	0.00	0.057	1.54	0.05	1.67	0.04
Fetal sonography at the time of amniocentesis										
Head circumference (mm)	−0.081	−0.95	0.01	−0.22	0.00	0.042	0.08	0.02	−0.23	0.02
Abdominal circumference (mm)	−0.089	−2.18	0.03	−1.14	0.00	−0.098 *	0.31 *	0.15 *	0.49	0.09
Femur length (mm)	−0.010	−3.63	0.01	19.39	0.05	0.157	0.32	0.02	−0.635	0.01
Estimated fetal weight (grams)	−0.067	−0.43	0.02	0.12	0.00	−0.002	0.06	0.10	0.07	0.02
Placental sonography at the time of amniocentesis										
Placental volume (mm^3^)	−0.044	0.15	0.01	0.10	0.00	−0.162	−0.01	0.00	−0.00	0.00
VI	−0.103	4.02	0.01	4.16	0.01	0.148	−0.10	0.00	−0.02	0.00
FI	−0.227	−0.60	0.01	−0.79	0.01	−0.161	−0.05	0.01	−0.06	0.02
VFI	−0.210	1.18	0.00	−0.01	0.00	0.011	0.02	0.00	0.01	0.00

PP13: placental protein-13, BMI: body mass index, GA: gestational age, VI: vascularization index, FI: flow index, VFI: vascularization flow index. * *p* < 0.05; ** *p* < 0.001.

## Data Availability

The data can be made available by corresponding authors on request.
